# Supplementary material for: Lifestyle risk factors and metabolic markers of cardiovascular diseases in Bangladeshi rural-to-urban male migrants compared with their non-migrant siblings: A sibling-pair comparative study
Source: PLoS One. 2022 Sep 27;17(9):e0274388. doi: 10.1371/journal.pone.0274388 (PMC9514650; doi:10.1371/journal.pone.0274388)
Supplement: S1 File — (DOCX) [file pone.0274388.s001.docx]

**S1 File**

**Sampling Technique**

In Bangladesh, there are seven administrative divisions, and each division is divided into districts/zilas, and each district into subdistricts/upazilas. A rural area in the subdistrict is divided into Union Parishads (UP) and mouzas within an UP. At first, all 168 mouzas of Pirganj subdistrict were listed for the household survey. Among them 70 mouzas of more than 300 households were identified for random sampling. Considering the average number of adults per household is four (50) and the rate of migration is 11.3 per 1000 people (51), mouzas of at least 300 households covering approximately 1,200 people can yield 11-12 migrants. Thus, for a required sample of 173 pairs we would need to survey 15 mouza of 300 households. However, since we set several inclusion/exclusion criteria (e.g., migrants only to Dhaka, had full sibling [same biological parents] of the same gender and agreed to provide contact information of sibling migrant) we increased the number of selected mouza to 26 of at least 300 households of 10 unions. These mouzas were randomly selected using a random number table to represent the Pirganj subdistrict of Thakurgaon. All households of the randomly selected mouza were approached to identify rural-to-urban migrants. Coverage of all households in a mouza increased the cost-efficiency of the survey. When the migrant of the identified household was confirmed for participation, then one rural sibling of the same gender of migrant was invited to take part in the study.

Once a rural sibling provided the name and contact number of their sibling migrant to Dhaka, we created a list of potential migrant participants, which formed our sampling frame for the migrant study. We contacted each of the migrants to Dhaka from the list, confirmed their eligibility and obtained consent before enrolment. Reasons for non-enrolment were noted. If multiple same-gender siblings were available, the one closest in age was invited. Migrants were asked to attend Bangladesh University of Health Sciences (BUHS) in Dhaka for data collection. Research assistants interviewed rural participants in their homes.
